# Using Multi-Antenna Trajectory Constraint to Analyze BeiDou Carrier-Phase Observation Error of Dynamic Receivers

**DOI:** 10.3390/s21206930

**Published:** 2021-10-19

**Authors:** Chenyao Xiong, Qingsong Li, Dingjie Wang, Jie Wu

**Affiliations:** College of Aerospace Science and Engineering, National University of Defense Technology, Changsha 410073, China; xiongchenyao19@nudt.edu.cn (C.X.); liqingsong10@nudt.edu.cn (Q.L.); wujie_nudt@sina.com (J.W.)

**Keywords:** GNSS, dynamic receiver, error bounding, integrity monitoring, characteristic analysis

## Abstract

Appropriate cycle-slip and measurement-error models are essential for BeiDou carrier-phase-based integrity risk calculation. To establish the receiver’s measurement-error model, an accurate position reference of the GNSS antenna is fundamental for calculating the measurement error. However, it is still a challenge to acquire position references for dynamic BeiDou receivers, resulting in improper GNSS measurement-error models and unreliable integrity monitoring. This paper proposes an improved precise relative positioning scheme by adopting multi-antenna trajectory constraints for dynamic BeiDou receivers. The dynamic experiments show an obvious decline of 78.7%, at most, in the positioning failure rate of the proposed method, as compared with the traditional method. The position solutions obtained from the proposed approach are used as the reference to analyze the cycle-slip and measurement-error characteristics of the dynamic receiver. The field test results indicate that the cycle-slip rate decreases with the increase of signal-to-noise ratio (SNR), and cycle slipping obeys a positively skewed distribution that could be fitted by the Gaussian mixture model (GMM). On the other hand, the standard deviation of the carrier-phase measurement error is inversely proportional to SNR, and its distribution is characteristically fat-tailed, which could be fitted by the bi-normal model.

## 1. Introduction

The Global Navigation Satellite System (GNSS) can provide accurate position and velocity information, thus playing an important role in various vehicular applications such as autonomous driving, location-based services, unmanned delivery and aviation navigation. To further expand the GNSS applications, it is necessary to improve GNSS performance, including accuracy, integrity, continuity and availability. The Federal Aviation Administration (FAA) defined a global instrument approach guidance service for aircrafts called LPV-200, which requires high positioning accuracy. Moreover, the integrity risk required by LPV-200 is lower than 10^−7^ per approach [1]. Accurate GNSS positioning heavily relies on the carrier-phase observation, whose performance is highly affected by cycle-slip and measurement-error characteristics. As a result, it is necessary to determine proper (or conservative) cycle-slip and measurement-errors models, especially for GNSS integrity risk calculation.

As for cycle slips, many existing works focus on cycle-slip detection and repairing mechanisms. However, the effectiveness of these methods lacks long-term experimental demonstration. Li [2] and Chen [3] adopted short-term measurement data, and artificially added specific cycle slips to it to verify the effectiveness of the proposed method. Chen [4] used different methods on vehicular test data for about ten minutes each to verify the effectiveness of a robust, extended Kalman filter in cycle-slip detection. Yet, this could lead to an underlying failure risk of the proposed method for some cycle slips in actual situations. The integrity risk that is required, by LPV-200, to be met is equivalent to that of an aircraft performing 47 years of GNSS-based approach guidance during both day and night, and only one failure due to having missed GNSS alarm is allowed (assuming that an approach takes 150 s). Such an extremely small value cannot be simply demonstrated experimentally within several minutes. The experimental durations and types of manually added cycle slips, to date, may not be sufficient to represent the actual situation under the influence of long-term and various risks, thus, these detection methods may still risk missing alarms. It is still indispensable to test these methods with data measured over longer intervals and to analyze the probability and characteristics of cycle slips in order to calculate the integrity risk caused by cycle slips and missed alarms.

This has prompted extensive researches on the GNSS carrier phase measurement error modeling for both static receivers and low-cost dynamic receivers. For static GNSS receivers in field surveyal, the carrier phase measurement error terms can be properly modeled and compensated for using accurate positioning results by long-term static measurement, contributing to extremely high positioning accuracy (e.g., mm-level) [5,6,7,8,9,10,11,12]. Roland et al., used an ARIMA model and non-parametric spectral estimation method to calibrate high-rate GNSS observations, successfully detecting vibrations on the order of magnitude of 10 μm~0.1 mm [13]. Luis et al., proposed an improved, static and precise relative-positioning method by reducing hardware and multipath delays, specifically for GNSS-based distance metrics, which provide baseline references with sub-millimeter accuracy [14]. As for dynamic GNSS receivers, the main difficulty lies in determining the position references for moving trajectories. Many studies on the carrier phase measurement errors of dynamic antenna have focused on low-cost GNSS receivers, using the position results from high-accuracy geodetic receivers as references for moving trajectories [15,16,17]. Li Guangcai et al., compared Android devices (i.e., Galaxy S8, Honor V8 and Nexus 9) with u-blox receivers and geodetic receivers and analyzed the pseudorange and carrier-phase error characteristics of the low-cost receivers on Android devices under static and dynamic conditions [18]. Chen et al., indicated that the differences between the pseudorange and carrier-phase observations of some devices are not fixed, by comparing different devices [19]. Gao et al., have pointed out that the integer property of the carrier phase ambiguity should be restored by a detrending operation [20]. Different from these low-cost GNSS receivers, the reference trajectories of high-precision receivers often need more precise instruments, which are usually difficult to deploy in dynamic conditions.

To obtain GNSS measurement errors in dynamic conditions, accurate position references at each time epoch should be acquired for dynamic GNSS receivers. Lau Lawrence et al., studied the GNSS multipath effects of dynamic receivers by conducting railway experiments [21]. The reference trajectories in the examined railway were precisely measured before the experiment, however, this experiment actively introduced multipath errors, such that accurate positioning results were difficult to determine. Therefore, they used double difference residuals as the reference standard for calculating multiple paths. Quan et al., used a GNSS receiver fixed on a slowly rotating metal rod to acquire GNSS observation data, while a total-station instrument was employed to provide precise synchronous observations, to aid in the evaluation of measurement error in their moving condition [22]. However, employing a total-station instrument restricts the dynamic range of GNSS receivers (i.e., antennae) to a relatively low rate (i.e., 0.21–0.72 m/s). Consequently, some GNSS measurement error terms cannot be fulfilled. In addition, the period of the observation data is too short to reveal the error characteristics thereof.

Motivated by the idea that mechanical structural constraints can be used to predict trajectory references, this paper proposes an improved precise relative positioning scheme by adopting multi-antennae trajectory constraints for dynamic BeiDou receivers to analyze carrier phase-error characteristics. In this scheme, four GNSS antennae are fixed on the same rotating platform, thus forming a geometric constraint, to collect GNSS observation data over longer times. At the same time, this structural constraint is used to derive a new constrained relative positioning model, based on double-differenced carrier phase observations between satellites and epochs. The accurate position solutions obtained from the proposed method are used as trajectory references in analyzing BeiDou carrier phase cycle-slip and measurement errors corresponding to each satellite in each epoch. The relationships between carrier phase cycle slipping or measurement error and satellite orbits and SNR are also analyzed in detail. The proposed method is then evaluated by actual long-time dynamic GNSS observations.

The structure of this paper is as follows. Section 2 devises the constrained, precise relative positioning model for dynamic GNSS receivers, and then presents our method for determining carrier-phase cycle-slips and measurement error. The experiment’s results and conclusions are given in Section 3 and Section 4, respectively. The contribution of this paper is the provision of an effective means of analyzing carrier phase-measurement error characteristics, which could be beneficial to carrier phase-based integrity monitoring.

## 2. Determination Method of Carrier Phase Cycle-Slip and Measurement Error

### 2.1. Experimental Scheme Design

Accurately positioned GNSS antennae, at each measurement epoch, are needed to determine carrier-phase cycle-slips and measurement error. Moreover, it operates automatically, instead of relying on manual intervention, which favors dynamic data collection with longer sampling durations. With consideration for changing weather, it was also necessary to forgo the use of some sophisticated instruments, such as total-station instruments, which would otherwise restrict the dynamic range and duration of measurements during field testing.

**Figure 1:**
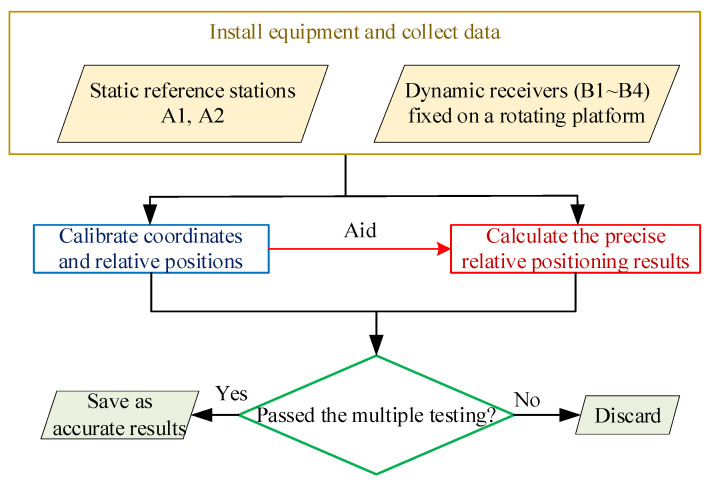
The main experimental steps (A means static antennae, B means dynamic antennae).

**Figure 2:**
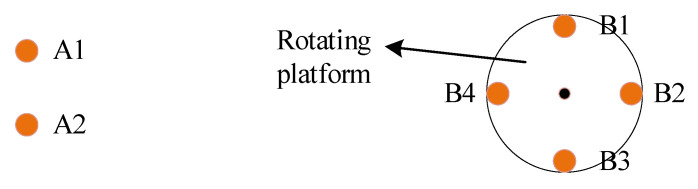
Installation diagram of antennae and rotating platform (A means static antennae, B means dynamic antennae).

**Figure 3:**
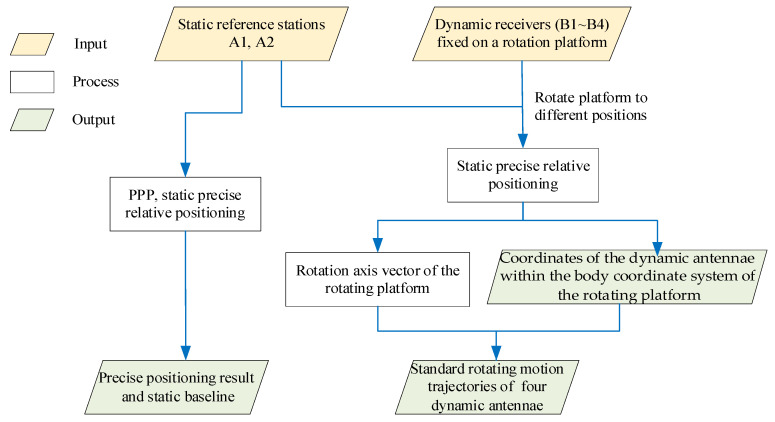
Procedure for calibrating coordinates and relative positions.

**Figure 4:**
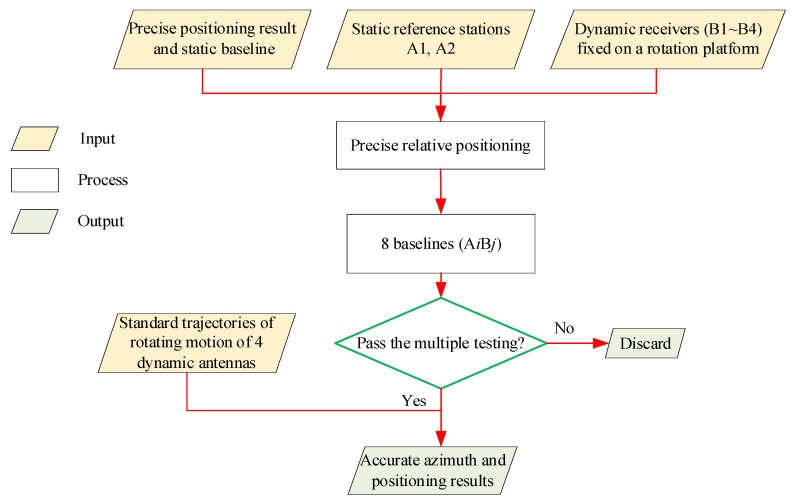
Calculate the valid precise relative positioning results.

As shown in Figure 1, the experiment can be divided into four modules:

(a) Installing the equipment and collecting data. Install four antennae on a rotating platform of the same radius as the dynamic antennae, at intervals of 90° (as shown at the top of Figure 2), and installed two static antennae not far away. Collect data as needed for calibration and in the calculation of dynamic results.
(b) Calibrating coordinates and relative positions. Calibrate the exact position of the static antennae and the relative positioning relationships between the dynamic antennae, and then calculated the motion trajectories of the antennae. After obtaining the above parameters, set the platform set to rotate at a uniform speed and began collecting data. The calibration method and contents are shown in Figure 3.
(c) Calculating precise and effective relative positioning results. Calculate the relative position of each dynamic antenna relative to the static antennae according to the collected data. In addition, since the orientation of the rotation axis of the rotating platform is fixed, when the body coordinate system of the rotating platform is defined, the real position of all dynamic antennae can be described by an azimuth parameter (similar to yaw angle). The schematic diagram is shown in Figure 4.
(d) Multiply test to ensure the accuracy of the positioning results. Error analysis requires sufficiently accurate positioning data as reference. Even for ultra-short baselines, there will be some epochs without a solution or with a wrong solution in long-term data, which is unfavorable to error analysis. In order to obtain reliable reference results, multiple testing is required for the positioning results at each time point.

**Figure 5:**
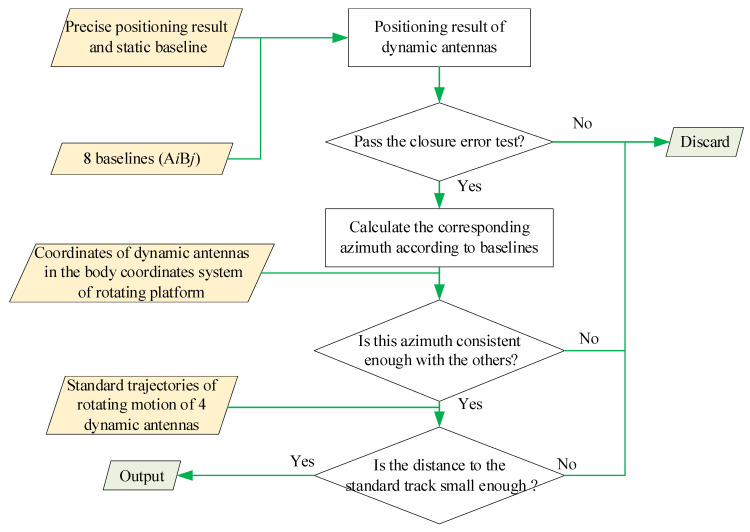
The contents of the multiple testing.

As shown in Figure 5, the contents of multiple testing include:

(i) Comparing the positioning results with antennae trajectories. If the deviation is too large (for example, horizontal deviation: >5 cm or the elevation deviation: >7 cm), we discarded it.
(ii) The relative positioning results from the dynamic antennae to the two static antennae need to be checked by a closure error test. Theoretically, the sum of the baseline vectors from the dynamic antenna to the two static antennae and the baseline vector of the two static antennae should be zero. Therefore, the position of the dynamic antennae can be considered accurate only when the sum of the three baseline vectors is lower than the given threshold in three-dimensional space (for example, 3D threshold = 8 cm).
(iii) According to the positioning results, the azimuth of platform rotation can be calculated, and the positioning results corresponding to the azimuth with excessive deviation should be discarded.

### 2.2. Using a Multi-Antennae Trajectory Constraint to Improve the Success Rate of Precision Relative Positioning in the Post-Processing Mode

The calculation of position reference depends on the correct ambiguity resolution. However, abnormal GNSS observation may lead to integer ambiguity resolution failure, thus failing to provide position reference at this time for cycle-slip and carrier-phase error analysis of the dynamic antennae. In order to overcome this limitation and improve the success rate of precise relative positioning (ideally, the location success rate should exceed 99.9% to reduce the impact of fault conditions on error modeling), two improvements for this experiment are proposed, as presented in this section.

#### 2.2.1. Geometric Constraints Aided Ambiguity Searching

Theoretically, if the initial position values of the two GNSS antennae are more accurate when the double-differenced carrier phase equation between antennae and satellites is established, the float solution of integer ambiguity will be closer to the correct value, and the success rate of subsequent least-squares ambiguity decorrelation adjustment (LAMBDA) search for integer ambiguity will be improved [23,24,25]. Considering that the dynamic antennae in this experiment move along a circular trajectory, this geometric constraint can be used to assist the ambiguity search when establishing the double-differenced carrier-phase equation between antennae and satellites.

Assuming a static reference antenna as *A*, a dynamic antenna as *B*, and the trajectory center of *B* as *O*, then the accurate coordinates of *A* and *O* can be obtained by static positioning. Thus, the position of dynamic receiver *B* can be expressed as:

(1) $${\vec{r}}_{B}={\vec{r}}_{A}+{\vec{r}}_{AO}+R_{n}^{e}\cdot R_{b}^{n}\cdot {\vec{r}}_{OB}^{b}$$

where *e* is the earth-centered earth-fixed (ECEF) frame; *n* is the north–east–down (NED) frame at the reference point *O*; *b* is the body frame of the platform, and the position vector of *B* can be referenced in the body frame as ${\vec{r}}_{OB}^{b}={\left[\begin{array}{ccc} x & y & z \end{array}\right]}^{T}$; $R_{b}^{n}$ is the transformation matrix from the body frame to the NED frame. Since the rotation axis of the turntable is perpendicular to the local horizontal plane, it can be considered that $R_{b}^{n}=R_{z}\left(\alpha \right)$, where $R_{z}$ is the rotation matrix around the *z*-axis, and the *z*-axis points skyward; $R_{n}^{e}$ represents the rotation matrix from the NED frame to the ECEF frame, which can be calculated as:

(2) $$R_{n}^{e}={\left(R_{e}^{n}\right)}^{T}={\left(R_{y}\left(-\left(B_{O}+\frac{\pi }{2}\right)\right)\cdot R_{z}\left(L_{O}\right)\right)}^{T}$$

where *B_o_* and *L_o_* are the latitude and longitude of *O*. The operators $R_{y}\left(•\right)$ and $R_{z}\left(•\right)$ represent the rotation matrix around the *y*- and *z*-axis, respectively; then Equation (1) can be expressed as:

(3) $${\vec{r}}_{B}={\vec{r}}_{A}+{\vec{r}}_{AO}+R_{n}^{e}\cdot \left[\begin{array}{ccc} \cos\alpha & \sin\alpha & 0\\ -\sin\alpha & \cos\alpha & 0\\ 0 & 0 & 1 \end{array}\right]\cdot \left[\begin{array}{c} x\\ y\\ z \end{array}\right]={\vec{r}}_{A}+{\vec{r}}_{AO}+R_{n}^{e}\cdot \left(\left[\begin{array}{c} 0\\ 0\\ z \end{array}\right]+\left[\begin{array}{cc} x & y\\ y & -x\\ 0 & 0 \end{array}\right]\cdot \left[\begin{array}{c} \cos\alpha \\ \sin\alpha \end{array}\right]\right)$$

According to the Equation (3), the double-differenced pseudorange and carrier phase equations between antennae and satellites with geometric constraints can be obtained as:

(4) $$\left\{ \begin{array}{l} \phi_{AB}^{ji}=\frac{1}{\lambda }\cdot \left(R_{AB,0}^{i}-R_{AB,0}^{j}\right)+\frac{1}{\lambda }\cdot \left({\vec{A}}_{B,cs}^{i}-{\vec{A}}_{B,cs}^{j}\right)\cdot \left[\begin{array}{c} \cos\alpha \\ \sin\alpha \end{array}\right]+N_{AB}^{ij}+\varepsilon_{\phi ,AB}^{ji}\\ \rho_{AB}^{ji}=\left(R_{AB,0}^{i}-R_{AB,0}^{j}\right)+\left({\vec{A}}_{B,cs}^{i}-{\vec{A}}_{B,cs}^{j}\right)\cdot \left[\begin{array}{c} \cos\alpha \\ \sin\alpha \end{array}\right]+\varepsilon_{\rho ,AB}^{ji} \end{array}\right.$$

where *j* represents the reference satellite; $\phi_{AB}^{ji}$ is the double-differenced carrier phase observation between antennae (i.e., **A** and *B*) and between satellites (i.e., *i* and *j*); $\rho_{AB}^{ji}$ is the double-differenced pseudorange observation between antennae (i.e., **A** and *B*) and between satellites (i.e., *i* and *j*); $R_{AB,0}^{i}$ is the single-differenced distance between the initial positions of **A** and *B* for the satellite *i*, i.e., $R_{AB,0}^{i}=\left|{\vec{r}}^{i}-{\vec{r}}_{A}\right|-\left|{\vec{r}}^{i}-{\vec{r}}_{O}\right|$; ${\vec{r}}_{A}$ represents the known position of static reference **A**; *λ* is the wavelength of the satellite signal wavelength; $N_{AB}^{ij}$ is the double-differenced integer ambiguity between antennae and between satellites; *ε* is the observation error; *α* represents the unknown azimuth of the rotating platform; and ${\vec{A}}_{B,cs}^{i}$ is the direction vector from point *O* to satellite *i*, which can be calculated as:

(5) $${\vec{A}}_{B,cs}^{i}=-\frac{{\left({\vec{r}}^{i}-{\vec{r}}_{B,0}\right)}_{}^{T}}{\left|\left({\vec{r}}^{i}-{\vec{r}}_{B,0}\right)\right|}\cdot R_{n}^{e}\cdot \left[\begin{array}{ll} x & y\\ y & -x\\ 0 & 0 \end{array}\right]$$

where ${\vec{r}}^{i}$ is the position of the satellite *i* at observation time, and ${\vec{r}}_{B,0}$ is the initial position value of antenna *B*. They are vectors of dimension 3 × 1.

By solving the Equation (4), $\sin\alpha^{\prime }$, $\cos\alpha^{\prime }$ and the float solution of double-differenced ambiguity can be obtained. It should be noted that the computed $\sin\alpha^{\prime }$ and $\cos\alpha^{\prime }$ are usually inaccurate and cannot meet ${\left(\sin\alpha^{\prime }\right)}^{2}+{\left(\cos\alpha^{\prime }\right)}^{2}=1$. The obtained ambiguity float solution and variance matrix can be used in the conventional LAMBDA search for the integer solution. It is anticipated that the geometric constraint can help reduce the number of equation unknowns and improve the ambiguity search performance especially when the number of available satellites is small.

#### 2.2.2. Interpolation Calculation and Secondary Processing

After checking the baseline vectors as described in Section 2.1, the accurate position solutions of each dynamic antenna can be obtained.

Considering that the platform rotation speed is relatively stable, it is assumed that the second derivative (angular acceleration) of the platform azimuth should be first-order differentiable. Moreover, the velocity derived from Doppler observations is accurate. As a result, the cubic spline interpolation can be performed for the platform azimuth to compute the missing position of the dynamic antenna.

After the interpolation calculation, in order to reduce the number of unsolvable epochs further, carry out secondary processing according to the following steps:

(a) Take the interpolated antenna position as the initial position, and then solve the float ambiguity calculation equation.
(b) Use LAMBDA to search the fixed ambiguity solution. As the initial value is more accurate, the success rate of this step will increase.
(c) Integrate the results of other antennae to obtain the accurate position.

**Figure 6:**
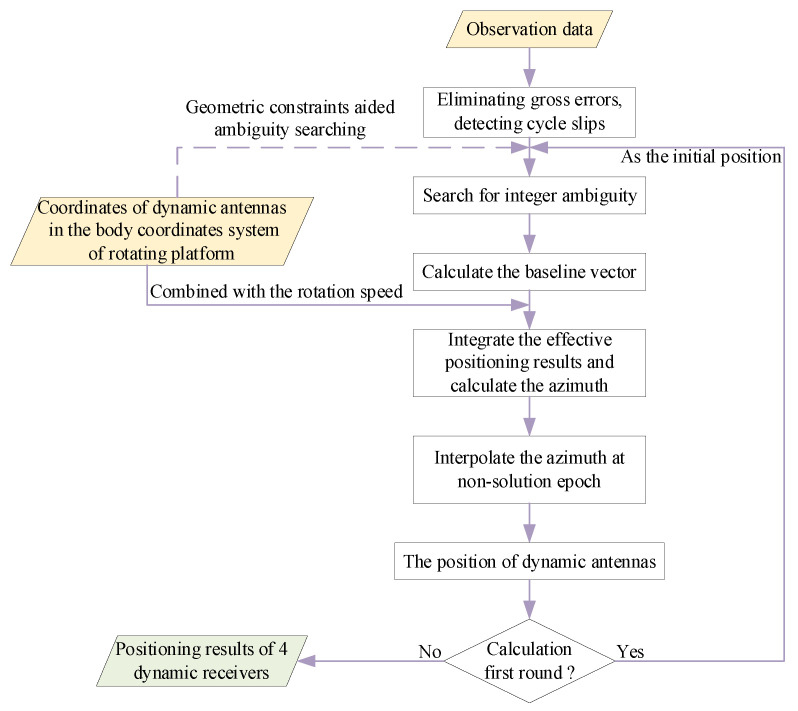
The method of improving the success rate of precise relative positioning.

The schematic diagram is shown in Figure 6.

### 2.3. Using the Mixture of Gaussian Distribution to Model Carrier-Phase Cycle Slips

At present, all cycle-slip detection methods cannot guarantee that they will never miss the alarm. Therefore, analyzing the characteristics of cycle slip is conducive to achieving both the integrity and availability indicators at the same time. Cycle slips are characteristically have sharp peaks and heavy tails, and do not comply with the normal distribution. However, it is also improper to fit its statistical characteristics using the skew-normal distribution model by experience. This paper proposes to fit its probability density with the mixture of gaussian (MOG) distribution as follows:

(6) $$p_{MOG}(x)=\sum_{j=1}^{k}P_{j}\left[\frac{1}{{\left(2\pi \right)}^{d/2}{\left|\Sigma_{j}\right|}^{1/2}}\exp\left(-\frac{1}{2}{\left(x-\mu_{j}\right)}^{T}\Sigma_{j}^{-1}(x-\mu_{j})\right)\right]$$

where *k* is the number of sub-Gaussian distributions in the mixture of Gaussian distribution, *P_j_* is the probability of the *j*th sub-Gaussian distribution, *μ_j_* is the mean value of the *j*th sub-Gaussian distribution, Σ*_j_* is the covariance matrix of the *j*th sub-Gaussian distribution, and *d* is the dimension of the variable ***x*** [26].

### 2.4. Using the Bi-Normal Distribution to Model Carrier Phase Measurement Error

It has been demonstrated that carrier phase measurement error does not fully comply with the Gaussian distribution. The actual measurement errors usually have fat tails [27], which undermines the effectiveness of the carrier phase-based receiver autonomous integrity monitoring (CRAIM) [28,29]. Compared to the Gaussian distribution, the bi-normal distribution can envelope the fat tails while retaining its characteristic spikes [30]. Through simulation experiments, Song [30] confirmed the effectiveness and robustness of the bi-normal distribution in integrity monitoring. Different from the mixture of Gaussian distribution, the bi-normal distribution is not the superposition of two Gaussian distributions, but the splicing of two Gaussian distributions after truncation. The error *ε* of this distribution is considered to be zero-mean and symmetrically distributed. In addition, the bi-normal distribution distinguishes between normal measurement and measurement fault in the formula, and introduces fault probability (*P_f_*), which helps to make the integrity monitoring more accurate or conservative.

**Figure 7:**
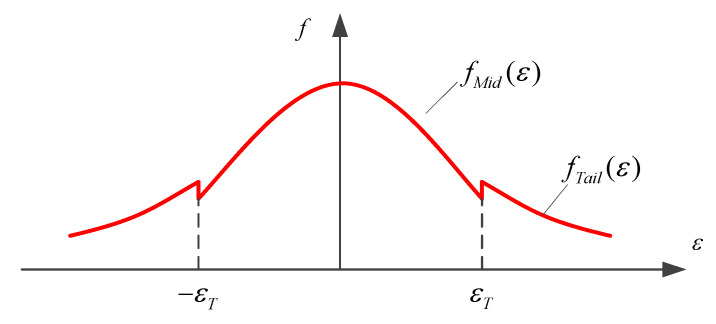
Diagram of bi-normal distribution.

The error within the threshold ±$\varepsilon_{T}$ obeys the Gaussian distribution of a small normal standard deviation $\sigma_{0}$, while the error outside the threshold obeys the Gaussian distribution of a large standard deviation $\sigma_{1}$. The probability density function schematic diagram is shown in Figure 7. $f_{Mid}(\varepsilon )$ and $f_{Tail}(\varepsilon )$ are defined as:

(7) $$\left\{ \begin{array}{l} f_{Mid}(\varepsilon )=k_{0}\cdot \frac{1}{\sqrt{2\pi }\sigma_{0}}e^{-\frac{\varepsilon^{2}}{2\sigma_{0}^{2}}}\;\varepsilon \in \left[-\varepsilon_{T},\varepsilon_{T}\right]\\ f_{Tail}(\varepsilon )=k_{1}\cdot \frac{1}{\sqrt{2\pi }\sigma_{1}}e^{-\frac{\varepsilon^{2}}{2\sigma_{1}^{2}}}\;\varepsilon \in \left(-\infty ,-\varepsilon_{T}\right)\cup \left(\varepsilon_{T},+\infty \right) \end{array}\right.$$

in which *k*_0_ and *k*_1_ are the parameters to adjust the curve shape to ensure that the sum of probabilities is 1.

This paper presents a method of calculating the parameters of the “bi-normal distribution” model. The probability of outlying error is *P_f_*, and the quantile is 1 − α = 0.5 × *P_f_*. For the standard normal distribution with the upper quantile *x* corresponding to *P_f_*, the standard deviation of the distribution for outlying error is $\sigma_{1}=\varepsilon_{T}/x$. In order to conservatively bound large measurement errors, a scaling factor *k* is introduced. Note the maximum occurrence frequency of an error within ±$\varepsilon_{T}$ in the measured data is *p*, it is required that:

(8) $$\begin{array}{l} f_{Mid}\left(\varepsilon_{T}\right)\geq k\cdot p\\ f_{Mid}\left(\varepsilon \right)=\frac{\left(1-P_{f}\right)\cdot \phi \left(\varepsilon ;0,\sigma_{0}\right)}{\Phi \left(\varepsilon_{T}\right)-\Phi \left(-\varepsilon_{T}\right)} \end{array}$$

where ϕ(·) represents the probability density function of the normal distribution; Φ(·) represents the normal distribution function, and the value is calculated by the integral of ϕ(·). The analytic solution of the abovementioned inequality is difficult to obtain, and the minimum $\sigma_{0}$ satisfying Equation (8) can be taken as the standard deviation of the normal distribution through the search method.

## 3. Experimental Results and Analysis

The experiment was conducted on the roof of a building in Changsha, China, and there were no buildings or trees above an elevation of 20°. Four dynamic antennae and receivers were fixed on a horizontal rotating platform with a radius of about 0.5 m. During the experiment, the rotation speed reached approximately 120 rpm, and the linear speed of the GNSS antennae about 6.3 m/s. The distances between the dynamic antennae and the static reference antennae were about 20 m, forming ultra-short baselines. The baseline length of the two static reference antennae was about 1 m. The GNSS sampling rate was 2 Hz. The B1I and B3I signal components of the BeiDou satellite navigation system L1C and the L2W signal components of GPS were collected during the experiment.

**Figure 8:**
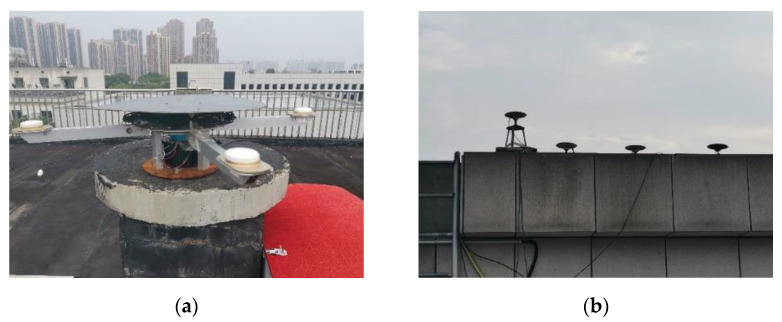
The experimental scene and equipment. (**a**) The rotating platform and four dynamic receiver antennae. (**b**) Static receiver antennae.

It is worth mentioning that the horizontal acceleration direction of the dynamic receiver changed rapidly during the experiment, which is definitely worse than in common application. The ComNav K708 OEM boards and Harxon HX-CA7606A aviation antennae were adopted for all four dynamic receivers and antennae, which can operate stably under such working conditions. The experimental scene and equipment are shown in Figure 8.

Carrier phase observations included receiver-clock error, ionospheric delay, tropospheric delay, satellite ephemeris error, multipath error [31], hardware noise [32], etc. Considering the beneficial factors such as fixed and known platform position, the short-time interval between two observations, and the inactive ionosphere during the experiment, the double-differenced carrier phase between epochs and satellites can eliminate most of the systematic errors but for cycle slips, and also amplify noise. Therefore, the double-differenced carrier phase residuals at each epoch can be decomposed into cycle slips and measurement errors by simple rounding.

### 3.1. The Effect of Geometric Constraints-Aided Ambiguity Searching

The method of geometric constraints-aided ambiguity searching reduces the number of unknowns in the equations, and uses a relatively accurate initial position, which can make the ambiguity float solution closer to the correct solution and improve the ambiguity-searching efficacy when the number of available satellites is small.

The limitation of the proposed method lies in its dependence upon the unknowns sinα and cosα, which violates the basic assumption of least-squares estimation. In addition, the positioning error in the elevation direction is also ignored, so that the residuals in the elevation direction are projected to the horizontal direction, which has a negative impact on the calculation of float ambiguity and its variance estimation. When the number of satellites is large, enough redundant satellites make the float ambiguity accurate enough, and the error-of-variance matrix reduces the success rate of searching ambiguity.

In this experiment, when only GPS satellites (less than nine) were observed, the adoption of the proposed algorithm witnessed a slight increase in the success rate of fixed solutions compared with the traditional method. However, when there were more than 20 satellites available, the performance of this method had no advantage over the traditional method except for calculation time.

Using the method in Section 2.2.2, the position of each epoch in the experiment has been correctly calculated, and these positioning results are used as reference positions. When the 3D error between the positioning result and the reference result of the geometric constraint method or traditional method does not exceed 7 cm, the epoch is considered to be solved successfully. The experimental results are shown in Table 1.

### 3.2. The Result of Interpolation Calculation and Secondary Processing

A 25-day corpus of GNSS observation data (about 4,327,810 epochs) was collected from 19 November to 14 December 2020. The number of effective epochs obtained by the above steps is shown in Table 2.

In this table, A*i* is a static reference antenna and B*i* is a dynamic antenna.

It can be seen from Table 2 that the interpolation calculation and secondary processing reduce the proportion of positioning-failure epochs from 1.99‰ to 0.56‰, a decrease of 72%. Compared with the traditional method checked by closure error test, the positioning failure rate of the new method is reduced by 78.7% at most. The risk of cycle-slip and error-characteristics analysis caused by positioning-failure epochs can be reduced as much as possible.

### 3.3. Cycle-Slip Characteristics of the Dynamic Receiver

#### 3.3.1. The Distribution Characteristics of Cycle Slips

**Figure 9:**
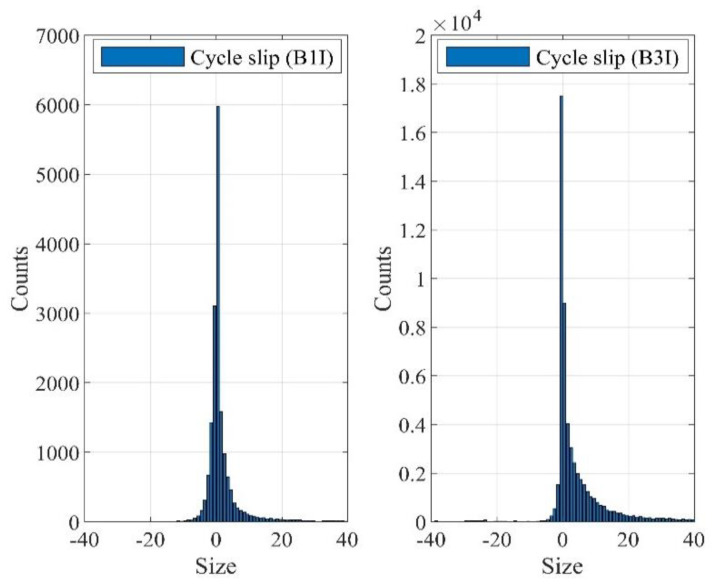
The histogram of cycle slips at B1I and B3I.

In this subsection, we explore the conditional distribution of the cycle-slip size when cycle slip has occurred, which could be conservatively approximated for subsequent assessment of cycle slips’ missed detection rate. It is observed that the distribution of cycle slips above ±40 cycles is relatively isolated. Therefore, cycle slips within (−∞, −40 cycles] and [+40 cycles, +∞) are counted together. The histogram of cycle slips at B1I and B3I signals of one antenna is shown in Figure 9.

It can be seen from Figure 9 that the incidences corresponding to different cycle-slip sizes do not obey the normal distribution. For cycle slips within (−40 cycles, +40 cycles), the kurtosis of the cycle slips at the B1I signal is 40.942 and the skewness is 5.884; while the kurtosis of cycle slips at the B3I signal is 29.970 and the skewness is 5.033. Obviously, the distributions of these two signals are skewed.

**Figure 10:**
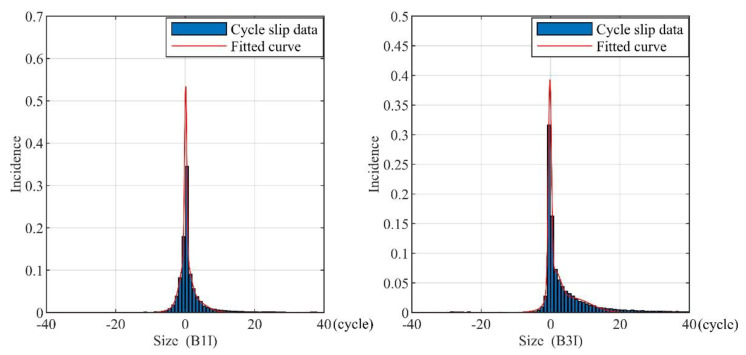
The fitting envelop curve of the cycle-slips histogram with the mixture of Gaussian distribution.

For cycle slips within (−40 cycles, +40 cycles), in this paper, a cubic Gaussian distribution mixture is adopted to fit the envelop curve of the cycle-slips histogram in Figure 10. The fitting formula can be expressed as:

(9) $$\left\{ \begin{array}{l} p_{MOG,B1I}=0.4070\exp\left(-{\left(\frac{x-0.1393}{0.4605}\right)}^{2}\right)+0.0985\exp\left(-{\left(\frac{x-0.0193}{2.0952}\right)}^{2}\right)+0.0326\exp\left(-{\left(\frac{x-16599}{5.2510}\right)}^{2}\right)\\ p_{MOG,B3I}=0.3441\exp\left(-{\left(\frac{x+0.1995}{0.6311}\right)}^{2}\right)+0.0523\exp\left(-{\left(\frac{x-1.2589}{2.3785}\right)}^{2}\right)+0.0248\exp\left(-{\left(\frac{x-5.9598}{7.5597}\right)}^{2}\right) \end{array}\right.$$

The fitting results are shown in Figure 10, and the statistical analysis of cycle slip data is listed in Table 3, which reveals that cycle slips occur differently at different sizes. Specifically, positive cycle slips are more likely to occur than negative ones, and small cycle slips are more likely to occur at all.

#### 3.3.2. The Relationship between Cycle-Slip Incidence and SNR

**Figure 11:**
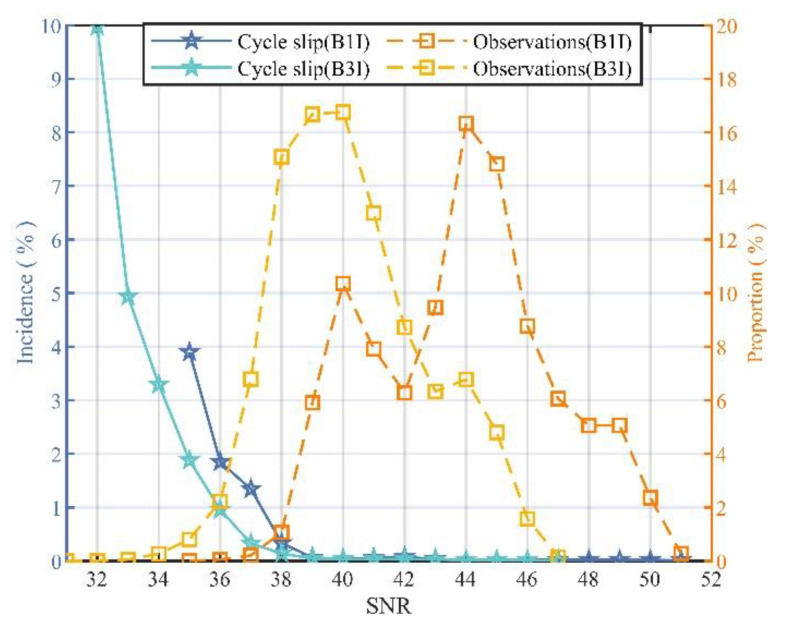
The change trend of cycle-slip frequency and measurement observations with SNR.

The signal-to-noise ratio (SNR) is observed by the receiver for carrier-phase changes, along with many factors such as weather, satellite positions, and environmental electromagnetic noise, etc. Figure 11 shows the trend of the cycle slips with SNR at B1I and B3I signals, using solid lines. Figure 11 also shows that the proportion of carrier phase observations at theB1I and B3I signals changes along with different SNRs using the dotted lines.

It is indicated that the cycle-slip probability decreases with the increase of SNR at both B1I and B3I. The weaker the signal, the easier it is for the receiver to lose lock and cycle slip. When the SNRs of B1I and B3Iis are larger than 34 and 31, respectively, the number of observations was over 1000.

**Figure 12:**
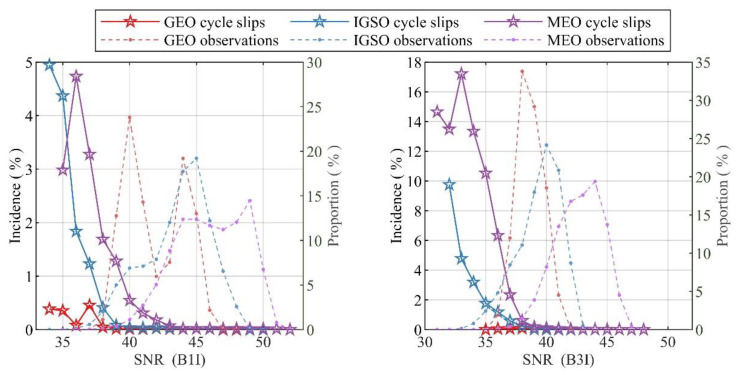
The relationship among cycle-slip incidence, SNR, and orbit.

Considering that BeiDou satellites have three kinds of orbits (i.e., GEO, IGSO, and MEO), this section also analyzes the relationship between SNR and the cycle-slip probability in different orbits, as shown in Figure 12. Owing to the distance between GEO satellites and receivers being almost constant, the variations in the SNR and cycle-slip probabilities are stable and small. However, the frequency of cycle slipping for the MEO satellites becomes large when the SNR is small, while the cycle-slip frequency of MEO satellites becomes small when the SNR is large. The reason may be that MEO satellites’ elevation changes rapidly and the propagation distance of their signals in the atmosphere also changes rapidly. In general, the number of MEO observations is only 1/3 of that of the IGSO observations, and 1/4 of that of GEO observations.

### 3.4. Carrier-Phase Measurement-Error Characteristics Analysis

#### 3.4.1. Distribution of Carrier-Phase Measurement Error

**Figure 13:**
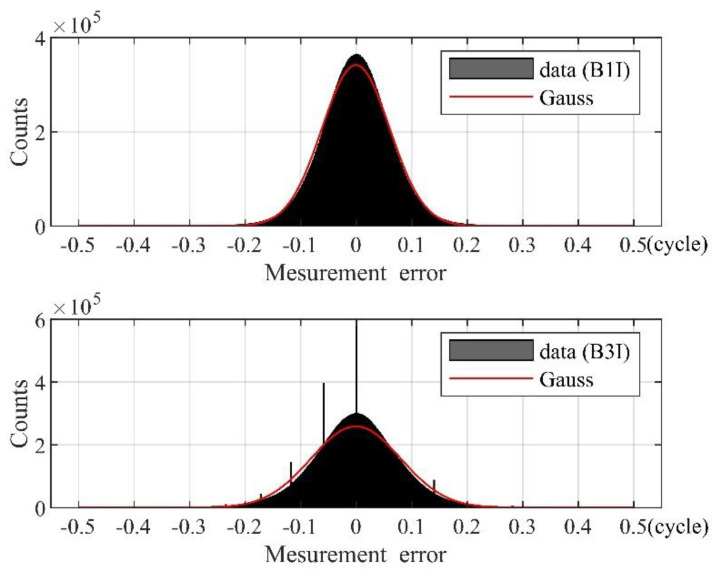
Fitting curve of the carrier-phase measurement error distribution and Gaussian distribution.

The average values of the carrier-phase measurement errors of the two frequencies are very close to zero. The standard deviation of the measurement errors at B1I was 0.061 cycles, and the standard deviation of measurement errors at B3I was 0.080 cycles. The normal distribution curve was fitted according to the average value and standard deviation, as shown in Figure 13.

It can be seen from Figure 13 that the carrier-phase measurement errors have an obvious peak and heavy tails for both the B1I and B3I signals. Usually, the measurement error with a deviation of more than 3σ from the mean can be regarded as the outlying error. Consequently, the outliers should account for 0.27% at each frequency for the normal distribution. Yet, the actual percentages of the outliers at B1I and B3I were 0.586% and 1.046%, respectively. This means that the actual outliers cannot obey the Gaussian distribution, and the thick tails should be depicted by bi-normal modelling.

Taking the measurement error on B3I as example, define k = 1.1, the bi-normal distribution density function at the B3I frequency is obtained as follows:

(10) $$f(\varepsilon )=\left\{ \begin{array}{ll} \phi \left(\varepsilon ;0,0.094\right) & \varepsilon <-0.2414\;\mathrm{or}\;\varepsilon >0.2414\;\\ 0.9940\times \phi \left(\varepsilon ;0,0.085\right) & -0.2414\leq \varepsilon \leq 0.2414 \end{array}\right.$$

**Figure 14:**
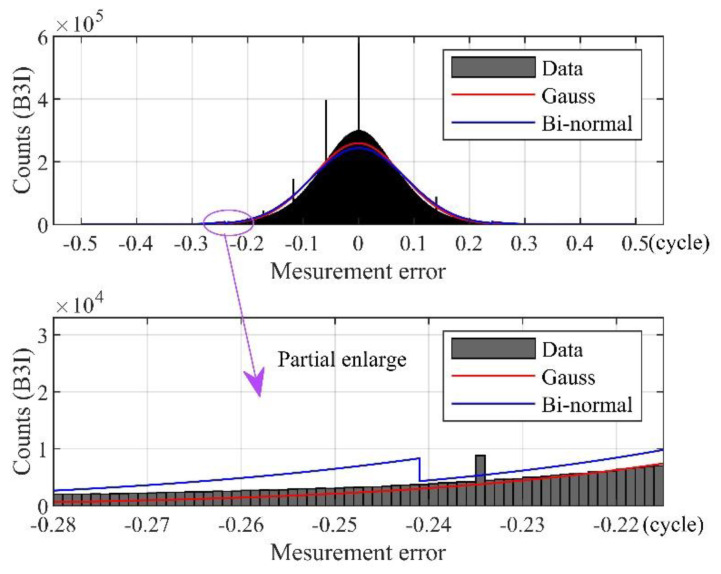
Envelope of bi-normal distribution.

The envelope curve of the bi-normal distribution is shown in Figure 14. It can be seen that the bi-normal distribution can bound the actual errors, showing better ability to bound the outliers than the Gaussian distribution.

Different receiver types, antennae types, satellite navigation systems, environments and dynamic conditions may lead to different measurement-error characteristics. The above model can be used as an example, and the parameters obtained are not necessarily universal.

#### 3.4.2. Relationship among Carrier Phase Measurement Error and Orbit

**Figure 15:**
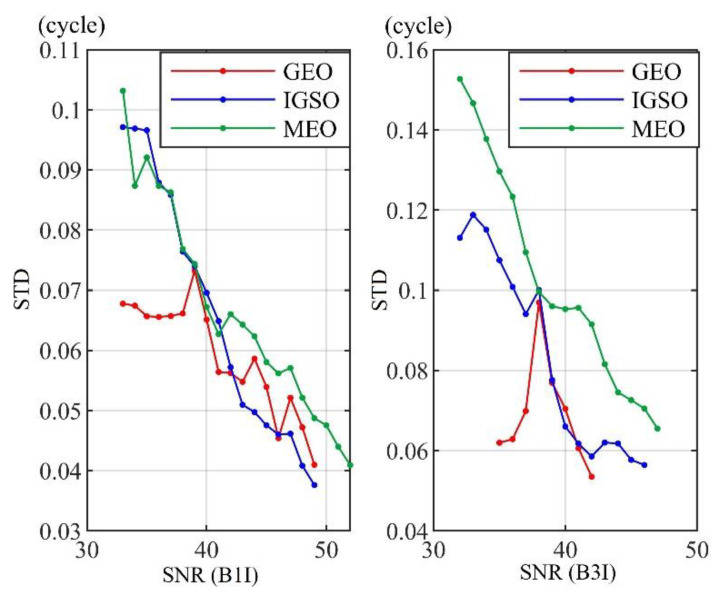
Variation of the carrier-phase measurement error STD with SNR and orbit.

When the different orbits are considered, the measurement errors of the two frequencies change along with SNR, as shown in Figure 15. As is general knowledge, with the increase of SNR, the error standard deviation (STD) of each orbit decreases. When the SNR is high, the standard deviation of the measurement error is relatively small. Moreover, the standard deviation of the measurement error of MEO satellites is larger than those of IGSO and GEO. A possible reason for this phenomenon may lie in transmission signal power differences among different satellites in different orbits. It has also been verified that the average signal power of high orbit satellites is not necessarily less than that of MEO satellites. The error standard deviation of IGSO and GEO is close, and IGSO is even better than GEO on the B1I frequency when the SNR is large enough.

## 4. Conclusions

To analyze the carrier-phase cycle-slip and measurement error of dynamic receivers, this paper proposes an improved precise relative positioning scheme by adopting multi-antenna trajectory constraints for dynamic BeiDou receivers. The long-term dynamic experimental results show that the new algorithm can decline the positioning failure rate by 78.7%, at most, using the techniques of interpolation calculation and secondary processing, as compared with its traditional counterpart.

With the acquired position as reference, it is feasible to analyze the cycle-slip- and measurement-error characteristics for a dynamic receiver. The dynamic test results indicate that the incidence of cycle slips decreases with the increase of signal-to-noise ratio (SNR), and cycle slips are more likely to happen in the observations of MEO satellites than in those of IGSO and GEO. On the other hand, the results also show that the carrier phase-measurement error distributions at B1I and B3I are similar to the Gaussian distribution, but have the characteristics of a sharp peak and thick tails. The standard deviation of carrier-phase measurement error correlates negatively with SNR, and large measurement errors are more likely to happen in the observations of MEO satellites than in those of IGSO and GEO ones.

In addition, it is indicated that both the carrier-phase cycle-slip probability and measurement error are larger than the empirical values, which could be explained, to some extent, by the fast-rotating condition of the GNSS receivers in the dynamic experiment.

## 5. Patents

The method described in Section 2.2.1 of this article has applied for an invention patent in China and has been authorized (CN112987038B).

## Figures and Tables

**Table 1:** The success rate of the geometric constraints method and the traditional method.

System	Solvable Epoch	Traditional Method	Geometric Constraints Method	Satellite Number (Min)	Satellite Number (Max)
GPS	9409	9108	9338	5	9
GPS + BDS	9414	9332	9310	21	27

**Table 2:** Number of effective epochs after each processing step.

Steps	A1B1/A2B1	A1B2/A2B2	A1B3/A2B3	A1B4/A2B4
Move to static precise relative positioning	4,319,319/4,319,644	4,319,688/4,320,046	4,318,656/4,319,033	4,318,900/4,319,280
Closure error test	4,316,937	4,316,677	4,316,478	4,316,870
Integrated processing	4,319,191			
Interpolation processing	4,324,975 (increased 5784 epochs)			
Secondary processing	4,325,669/4,325,803	4,325,595/4,325,786	4,325,634/4,325,800	4,325,577/4,325,723
Closure error test	4,324,550	4,324,531	4,324,575	4,324,383
Integrated processing	4,325,062			
Interpolation processing	4,325,400 (increased 338 epochs)			

**Table 3:** Size of cycle slips statistics.

Interval	B1I	B3I	Interval	B1I	B3I
≤−40	878	650	(0, 5)	9178	18,486
(−40, −35]	0	78	[5, 10)	1235	7541
(−35, −30]	0	67	[10, 15)	398	3565
(−30, −25]	5	221	[15, 20)	213	1887
(−25, −20]	4	150	[20, 25)	141	1173
(−20, −15]	10	110	[25, 30)	106	753
(−15, −10]	46	51	[30, 35)	43	636
(−10, −5]	348	229	[35, 40)	73	432
(−5, −0)	5523	19,781	≥−40	1563	4275

## Data Availability

Not applicable.
